# Applying a panel network formation model to limited partnership matching in the private capital market

**DOI:** 10.1007/s13278-025-01527-7

**Published:** 2025-12-15

**Authors:** Yuanyuan Shang, Philip Leifeld

**Affiliations:** 1 Preqin Ltd., 10 Bressenden Pl, London, SW1E 5DH UK; 2 Department of Social Statistics, University of Manchester, Humanities Bridgeford Street Building, Oxford Road, Manchester, M13 9PL UK

**Keywords:** Private capital, Alternative assets, Limited partners, General partners, Network formation, Temporal exponential random graph model (TERGM)

## Abstract

In private capital investment, limited partners (LPs) and general partners (GPs) frequently encounter the challenge of finding suitable counterparts amid limited information, a process often hindered by market inefficiencies. This article addresses this issue by exploring the micro-level mechanisms that shape private capital networks, employing temporal exponential random graph models. Our findings uncover activity and popularity effects, persistence mechanisms, and homophily in preferences concerning region, strategy, and industry. These factors jointly shape the dynamically evolving network structure across asset classes and the hybrid network with all asset classes, revealing a shared network formation process. This article offers practical insights into the matching problem within the private capital market.

## Introduction

Markets where transactions occur between diverse agents are prone to matching problems. The challenge is to identify an appropriate “assignment” in the configuration of partnerships (Koopmans and Beckmann 1957; Roth and Sotomayor 1992). The agents in these markets have specific preferences regarding their transaction partners and are in competition for a limited number of sought-after partners. In equity finance networks, inefficient matching leads to an economically inefficient distribution of equity finance among the firms (Mindruta et al. 2016). Among the matching problems involving various combinations of agents in corporate finance, this article focuses on general partners (GPs) and limited partners (LPs). Within limited partnerships, GPs, responsible for managerial decisions and bound by a fiduciary duty, manage the funds, set investment strategies, and handle liabilities. LPs, including institutional entities and wealthy individuals, provide capital, committing specific amounts over the fund’s lifespan. GPs raise these funds from LPs and manage them within a partnership structure. The relationships between GPs and LPs are facilitated through the creation of funds. Together, GPs and LPs form a private capital network, with GPs and LPs serving as nodes and the relationships between them—established through these funds—acting as ties.

However, LPs and GPs face a partnership matching problem characterised by noisy information, transaction costs, and the risk that committing to one mid-term opportunity may result in the loss of alternative investment prospects. Faced with these challenges, GPs and LPs scrutinise the market for suitable partners, looking for signals of trustworthiness, interest alignment, and strong performance—both directly from potential partners and from the wider network neighbourhood through structural considerations. To understand the formation of commitments between LPs and GPs, we analyse the network of the private capital market and examine the roles of relational effects like popularity, activity, and homophily in partnership matching.

Network analysis enables researchers to gain valuable insights into the structure, evolution, and behaviour of financial networks, shedding light on crucial aspects of economic systems, by visualising and modelling these networks. It has emerged as a powerful tool to decipher the underlying mechanisms governing financial relationships (Bardoscia et al. 2021). Network theory has been applied to investigate a variety of complex issues in the financial world. This includes analysing the architecture of the interbank market and credit market (e. g., De Masi 2009; Langfield et al. 2014), understanding the stock markets correlations (e. g., Gałązka 2011), exploring the dynamics and factors influencing international trade (e. g., Bhattacharya et al. 2008; Dong et al. 2018), and examining how financial crises can spread through different systems, leading to widespread contagion (e. g., Leventides et al. 2019; Summer 2013).

The private capital market comprises diverse asset classes, such as private equity or infrastructure, with distinct investment strategies. Understanding how the formation of investment relationships differs within specific asset classes and the entire market can provide a comprehensive view of the interconnectedness between different segments of the private capital landscape. Some studies have explored the investment networks of private capital, such as Sorenson and Stuart (2001) and Porter and Spriggs (2013), but most studies only focus on venture capital or private equity (e. g., Giovannetti and Pipic 2023).

Research on networks in other asset classes—such as private debt (e. g., Godlewski and Sanditov 2020; McCumber 2013), real estate (e. g., Boaventura et al. 2016; Wang and Xie 2015), natural resources, and infrastructure—is scarce. When it does exist, it tends to focus on management or governance networks rather than the dynamics of investment networks. This paper addresses this gap in the literature and models the mechanisms governing investment behaviours between GPs and LPs in private capital networks across different asset classes.

Furthermore, research has explained why some firms are more central in the network than others and demonstrated how network positions predict firm performance or survival (e. g., Hochberg et al. 2007 in the case of venture capital). But to understand centrality, connectedness, or other node outcomes as part of the network formation process, we need to explain why any two GPs and LPs are connected. Modelling GP–LP ties is also instrumental for understanding how the global structure of the private capital network is generated.

The dynamics of private capital networks are not only influenced by traditional economic factors like inflation, regulatory environment, and economic cycles but also by various investment preferences, such as strategy preferences (Zhong et al. 2018) and geographical preferences (Mason 2007). Although preferences play a vital role in guiding investment decisions, there is little research in the literature looking into how shared preferences concerning regions, strategies, and industries between GPs and LPs influence the structure of private capital networks. This study tries to fill this gap and understand the impact that shared preferences have on network formation in order to explain how investors allocate capital across different investment opportunities.

The primary objective of this research is to understand the generative mechanisms of investment networks in the private capital market by examining the investment networks of GPs and LPs from a longitudinal perspective. We capture the temporal changes in network structures and identify the factors that drive the formation of investment ties between GPs and LPs over time. Furthermore, we aim to investigate whether the generative mechanisms that drive investment networks in individual asset classes are the same as those driving what we call the “hybrid” network, which includes all asset classes.

To analyse the dynamics of private capital networks, this study employs the Temporal Exponential Random Graph Model (TERGM) (Cranmer and Desmarais 2011; Hanneke and Xing 2010; Leifeld et al. 2018), a statistical approach that captures how network structures evolve over time. We apply this model to a proprietary dataset from Preqin (part of BlackRock, Inc.), comprising annual networks of investment relationships between GPs and LPs. By leveraging this dataset and applying the TERGM methodology, we can offer a comprehensive network analysis of the private capital market.

Our research contributes to improving market efficiency by shedding light on how investments come about and shape the macro structure of private capital networks. We uncover co-commitment dynamics, emphasising interconnectivity and collaboration among financial actors in the private capital market. Co-commitments in this paper refer to investments where multiple LPs make commitments to funds managed by a single GP in a single year, without requiring commitments to be simultaneous or to the same fund.

Specifically, we examine how shared preferences–such as aligned regions, strategies, and industries–affect the likelihood of LPs investing in GPs. We find that co-commitment patterns are shaped by both similarity and differentiation among LPs, as well as network effects driven by past investment ties and the popularity or activity levels of GPs and LPs. These dynamics offer insights into how investment relationships form and persist in the private capital market.

The implications of this study are twofold: Firstly, for industry practitioners, the findings provide actionable insights and recommendations, aiding decision-making processes and potentially serving as a first step towards predicting future investments. Secondly, for academics, our research contributes to an understanding of the factors driving investment behaviours in the finance world, as it is grounded in empirical data, providing real-world observations and patterns. Furthermore, beyond its direct applications, our study also contributes to the field of network science. Notably, we apply the TERGM on relatively large bipartite networks, demonstrating the adaptability of this methodology to analyse complex financial networks.

## How LPs and GPs interact in the network

The private capital market is known to be more inefficient than the public market, stemming from various factors including information asymmetry, less stringent regulatory requirements relative to public markets, high transaction costs, and a heavy reliance on networks and relationships (Fenn et al. 1997; Wright and Robbie 1998; Spindler 2009). In this market, information is scarce, noisy, and highly valued (Alchian and Demsetz 1972; Pomatto et al. 2023). Information scarcity and power asymmetries further exacerbate these inefficiencies (Batt and Appelbaum 2021). While some LPs may hold limited voting rights or advisory roles, they often have constrained mechanisms to influence fund management or respond to underperformance, particularly compared to public market investors (Magnuson 2017). Unlike public market shareholders who can vote or exit their investments, LPs typically have no such mechanisms to influence management decisions or withdraw from underperforming funds. They may only exit under special circumstances or by selling their shares on the secondary market, which requires the fund manager’s consent and often involves “haircuts”—that is, a discount relative to the net asset value (NAV) (Shobe 2016). Moreover, GPs have significant control over fund management and performance reporting, which can obscure the true performance of investments due to practices like inflating NAVs and using flawed performance metrics such as the internal rate of return (IRR) (Jenkinson et al. 2013). This scarcity challenges GPs and LPs in identifying suitable counterparts, exacerbating the sorting or selection problem prevalent in private capital markets. GPs, particularly new entrants lacking a track record, invest significant time and resources to sift through numerous LPs and engage with them. Conversely, LPs face the costly task of seeking GPs who align with their interests and demonstrate the potential for strong performance. The key question arises: How can GPs and LPs make informed decisions with such limited information?

To address this challenge, it is essential to understand the role of networks within the private capital market. Networks serve a dual function, conceptualised as both “pipes” and “prisms” (Podolny 2001). As “pipes”, networks facilitate the flow of critical information and resources, enabling GPs and LPs to access the market intelligence necessary for making informed decisions. This information flow helps mitigate the effects of information asymmetry by providing insights into potential partners’ capabilities and reputations. Simultaneously, networks function as “prisms”, where the presence or absence of ties acts as a signal of quality and credibility to other market participants. This signalling is crucial in an environment where direct information is scarce, as it influences perceptions and trustworthiness, thereby guiding the decision-making process. By understanding these dual roles, we can better analyse how GPs and LPs navigate their relationships and how network positions can impact their success in forming profitable partnerships.

Here, we outline a set of hypotheses that serve as the foundation for our investigation into the dynamics of LP–GP interactions within the private capital investment network. These hypotheses are derived from both existing theoretical perspectives and empirical observations in the field.

First, we posit that there is a popularity effect, conceptualised as a structural tendency in the network. In statistical network modelling, popularity refers to the tendency for certain nodes—in this case, GPs—to attract multiple ties simply by virtue of already having many connections. This reflects the principle of preferential attachment, where nodes with higher degrees become more likely to gain additional links over time, independent of their specific attributes (Barabási and Albert 1999). In private markets, this structural tendency may manifest when LPs disproportionately commit to funds managed by GPs who already have many LP backers. Multiple LPs tend to make commitments to the funds managed by attractive GPs while funds managed by relatively unattractive GPs receive few or no commitments, leading to a clustering of LP ties around GPs. This process mirrors cumulative advantage or preferential attachment, frequently observed in social and economic networks. We evaluate this popularity effect by examining the clustering of LP ties around GPs, operationalised through the presence of “two-stars” in the network–configurations where multiple LPs are tied to the same GP. A higher-than-expected number of two-stars suggests popularity clustering.

Irrespective of the characteristics of the GP, this popularity effect is perhaps amplified by peer mechanisms. LPs observe the market and attribute a higher expected risk-adjusted return to a GP if other LPs also invest to the funds managed by the same GP. Doing so may help mitigate agency costs and reduce perceived investment risks. This dynamic aligns with social influence and information cascades as defined in the network and behavioural finance literature (Bikhchandani et al. 2024). Recent studies have shown that social influence can significantly impact asset prices, leading to complex dynamics in financial markets (Huang et al. 2023). Moreover, the structure of social networks plays a crucial role in decision-making processes, with certain network topologies facilitating more pronounced social influence (Zheng et al. 2024). In such models, agents take cues from the observed actions of others, particularly when private signals are noisy or costly to obtain. Like any investor, LPs face uncertainty over making the right choices to maximise returns. This imitation dynamic can also reduce agency costs and perceived risk, as LPs may feel more secure in following group behaviour. As shown in the finance literature, LPs may demonstrate herd behaviour, characterised by the emulation of their peers’ actions without conducting rigorous, independent due diligence (Chang et al. 2000; Hirshleifer and Hong Teoh 2003; Buchner et al. 2020). In such instances, the initiation of an investment by one LP can serve as a catalyst, setting off a cascade effect as others opt to follow the same path. Recent high-profile cases, such as the bankruptcies of Theranos and FTX (Allen 2022; Nurdiani 2023), underscore the real-world consequences of herd behaviour in investment decisions. These cases demonstrate that the popularity of certain GPs, driven primarily by herding and peer effects, can lead to significant financial risks. LPs should be mindful of these dynamics and the potential impact of herd behaviour when evaluating investment opportunities.

Multiple factors contribute to the attraction and popularity of GPs among LPs, including their track records and expertise. GPs who have consistently delivered attractive returns in the past are more likely to draw the interest of LPs. Furthermore, GPs with substantial industry knowledge, operational proficiency, and a proven capacity to enhance portfolio company performance are highly sought after and contribute to their popularity among LPs. Distinguishing empirically between attraction effects (stemming from GP qualities) and peer effects (stemming from social influence) as possible mechanisms underlying popularity clustering would require a near-perfect time ordering of investment observations (Malang et al. 2019). However, this is impossible to achieve in practice because the LP’s decision time and execution time of the commitments may not coincide, making it difficult to disentangle in which exact order commitments were decided upon. Moreover, the attractiveness of a GP for investment is merely a latent variable that is shaped by revealed behaviour by LPs, not a measurable quantity. If it were, market allocation would be efficient and deterministic. Hence, it is virtually impossible to disentangle latent attraction force and peer mechanisms underlying the aggregate popularity effect empirically. Without precise knowledge of the causal pathway, we resort to testing whether popularity clustering is present in the data and assessing how it contributes to aggregate network formation. To ensure conceptual clarity and alignment with our modelling approach, we therefore treat popularity in this context as a purely structural phenomenon, where a GP’s existing ties increase the likelihood of new ones, independent of their inherent qualities. We model this popularity effect by testing whether LP clustering around GPs exceeds what random tie formation would predict, keeping other network formation mechanisms constant. This leads to our first hypothesis below.

### H 1

Popularity: Multiple LPs tend to engage in investments with the same GPs.

Peer effects may initially reinforce popularity through social learning and signalling (Golub and Jackson 2010). However, once a threshold is reached—where a GP has drawn disproportionate attention—the marginal benefit of following the crowd may decline, giving rise to LP attention in other GPs. This inflection point can arise due to factors like limited fund capacity, diluted GP attention, or crowded informational signals. In such cases, rational LPs may begin to seek out less popular GPs with untapped potential, effectively “betting on underdogs” to access alpha—that is, excess returns beyond what is expected given the risk—not yet fully recognised by the market (Wei et al. 2017). This saturation point does not imply random allocation, nor does it necessarily lead to negative returns, but marks a zone where the benefits of herding taper off, prompting more strategic divergence in LP behaviour.

This theorised saturation mechanism provides conceptual grounding for our second hypothesis. As the marginal benefit of investing in highly popular GPs diminishes, LPs may rationally diversify their commitments across a broader set of GPs. This behavioural shift reflects a response to the declining informational and performance advantages of herding, and motivates our expectation that LPs will exhibit activity clustering by engaging with multiple GPs. Thus, while the popularity effect (H 1) captures the initial clustering of LPs around prominent GPs, the activity effect (H 2) reflects a complementary dynamic in which LPs seek diversification and potential alpha by allocating capital across a wider array of managers.

LPs value high risk-adjusted return, but they often avoid downside risk. In their pursuit of high risk-adjusted returns, LPs invest with multiple GPs in an attempt to diversify their portfolios (Kuhle 1987). Thus, investment activity by GPs clusters in the same LPs, irrespective of the GPs’ popularity. Activity clustering in LPs should hold both within and across different asset classes. We capture this tendency in the network model by counting two-stars centred around LPs and how much more often they occur than chance would predict in a random graph of the same size, controlling for other network formation mechanisms. Both popularity and activity are non-linear effects and act together with all other structural network effects in generating the observed network. The tapering off of GP popularity clustering is in part generated by LP activity and other structural effects as an equilibrium of network formation is reached.

### H 2

Activity: Individual LPs tend to diversify their investments across different GPs.

While private markets lack the transparency of public markets, they are not entirely opaque. LPs gather information through a range of imperfect but meaningful channels. Industry databases (e. g., Preqin, PitchBook), placement agents, LP advisory committees, syndication history, conference participation, and fund marketing materials all serve as signals of peer activity and manager quality. Through these mechanisms, LPs gain partial visibility into the behaviours, preferences, and fund selections of their peers–even if such information is delayed or incomplete. In addition to the structural features that emerge from the internal dynamics of the network—i. e., endogenous network effects where the presence or absence of ties depends on other ties—we posit that preference homophily between LPs and GPs shapes investment decisions and tie formation. In this paper, we focus on three key types of preferences that drive homophilous investment decisions: geographical, strategy, and industry preferences, as described in the following hypotheses.

### H 3

Preference homophily: LPs are more inclined to invest in funds managed by GPs who share common preferences with them.

Firstly, spatial patterns have been shown to be important for investments, especially when it comes to venture capital (VC). According to Cumming and Dai (2010), private equity investors, especially VC funds, are commonly seen as engaged investors who dedicate considerable time to participate on the boards of start-up companies. They offer valuable insights in areas like strategy, finance, marketing, and operations. These investors often maintain substantial authority, including the contractual ability to change the company’s CEO, and they usually have the power to veto decisions. As a result, it is reasonable to anticipate that these private equity investors would choose to invest in nearby start-ups. The close physical proximity of a VC investor to their target company is important, as frequent visits to assess the company’s operations are requested during the post-investments phase for oversight efforts (Chen et al. 2010; Sorenson and Stuart 2001). Existing evidence confirms that the local proximity between VC firms and investee companies is a critical determinant for the success of early-stage investments (e. g., Carlson and Chakrabarti 2007; Cumming and Dai 2010). Dupuy et al. (2010) found that large equity investors’ portfolio turnover is connected to geographical elements and their alignment with the specific capitalism model, that is, national or regional systems of capitalism which shape how investors engage with companies, including their investment turnover and preferences.

### H 3a

LPs tend to invest in funds managed by GPs who share common geographical preferences with them.

Secondly, LPs and GPs often seek alignment in their investment objectives and strategies. When LPs and GPs share common strategy preferences, it suggests a mutual understanding and alignment of interests, which can foster collaboration and trust (Freiburg and Grichnik 2012). This alignment is particularly vital in private equity, where the illiquidity and long-term nature of investments amplify the risks associated with agency problems. Steindl (2013) emphasises that misaligned interests—such as divergent time horizons, return expectations, or risk appetites—can lead to inefficiencies and reduced fund performance. To mitigate such risks, LPs increasingly assess the degree to which a GP’s investment philosophy aligns with their own, viewing strategic compatibility as a mechanism for reducing monitoring costs and improving performance predictability. Furthermore, investing with GPs who share similar strategy preferences allows LPs to better anticipate decision-making behaviours and portfolio exposures, thus serving as a form of implicit risk mitigation. For instance, Zhong et al. (2018) introduced a collaborative filtering model involving investment preferences among all investors. This model is designed to create personalised portfolio strategies, aiding investors in their pursuit of suitable start-up investments, which shows the importance of aligned strategy preferences for investments in private capital markets.

### H 3b

LPs tend to invest in funds managed by GPs who share common strategy preferences with them.

Thirdly, shared industry preferences also lead to homophilous tie formation in private capital networks. The market sectors of ventures play a pivotal role during the evaluation phase of investment deals (Tyebjee and Bruno 1984). This observation underscores the significance of industry expertise and preferences in investment decision making. Research shows that LPs view GPs’ prior experience and domain knowledge as indicators of fund quality and future performance (Zarutskie 2010). Consequently, LPs are more likely to allocate capital to GPs whose investment focus aligns with their own industry preferences, reinforcing patterns of similarity-based tie formation.

### H 3c

LPs tend to invest in funds managed by GPs who share common industry preferences with them.

Partial observability in private capital markets becomes especially relevant when considering competition and crowding effects. Even in the absence of full transparency, LPs can often infer fund demand and peer activity through indirect signals—such as publicised commitments by prominent institutions, investment announcements, or cues provided by placement agents and other intermediaries. We further expect to see niche formation in the investment landscape, as LPs with similar geographical and strategy preferences differentiate their investment behaviours to reduce competition. According to organisational ecology theory, entities operating in similar niches tend to engage in resource partitioning to avoid direct competition and ensure long-term survival (Carroll and Hannan 1989; Carroll and Swaminathan 2000). This logic extends to private capital markets, where LPs with overlapping preferences may deliberately avoid investing in the same funds to reduce crowding and preserve access to differentiated opportunities. For example, LPs may respond to limited fund capacity by intentionally diverging in their GP selections to avoid competing with peers for allocation slots. Moreover, studies of syndication and partner selection suggest that investors consider not only alignment but also the competitive exposure created by shared ties (Cox Pahnke et al. 2015). Once geographical and strategy matching between LPs and GPs is controlled for, LPs may choose to specialise in distinct investments, thereby reducing redundancy in their portfolios and minimising the risk of competing for limited fund allocations. In such cases, they are less likely to invest in funds managed by the same GPs to avoid competition and maintain access to a broader range of opportunities.

### H 4

Any two LPs who share the same geographical and strategy preferences are less likely to invest in funds managed by the same GPs, controlling for LP–GP geographical and strategy homophily.

These hypotheses provide a structured framework for our study, allowing us to estimate the dynamics of LP–GP interactions in the private capital investment network. We will empirically test these hypotheses to gain insights into the factors driving investment decisions and network formation in this ecosystem.

## Private capital investment data

Our study draws upon data sourced from Preqin, a leading provider of data and insights on the private capital industry. The data used in this research were acquired through a collaborative initiative between the academic institution of the authors and Preqin, with direct access granted to Preqin’s internal database. The dataset includes detailed information on investment relationships between GPs and LPs, as well as the characteristics of the involved entities and their investment preferences.

**Fig. 1:**
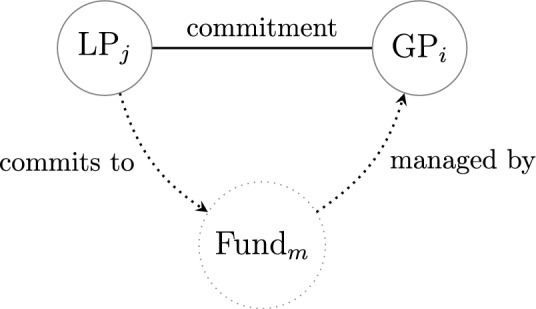
Illustration of the LP–GP connection through a fund. This paper models only the induced LP–GP network represented by the solid line.

We constructed annual, bipartite networks of GPs and LPs connected through funds based on the data. The dataset for our analysis covers the global private capital investments between LPs and GPs through funds from 2011 to 2021, including 12,719 LPs and 8,986 GPs in total across the years. The temporal data are based on the vintage years of funds, i. e., we assume the investments between an LP and a GP occurred in the year when the fund, which the GP manages and the LP invests in, was established. We constructed networks of GPs and LPs using commitment data, where GPs and LPs serve as nodes, and the financial commitments from LPs to the GPs managing the respective funds form the edges. For instance, when LP_j_ commits capital to Fund_m_, managed by GP_i_, LP_j_ and GP_i_ are represented as nodes in the network, with an edge connecting them, as illustrated in Fig. 1. Although individual ties have direction in the underlying investment process, the networks in our study are modelled as undirected to capture the reciprocal nature of the interactions between LPs and GPs.

**Fig. 2:**
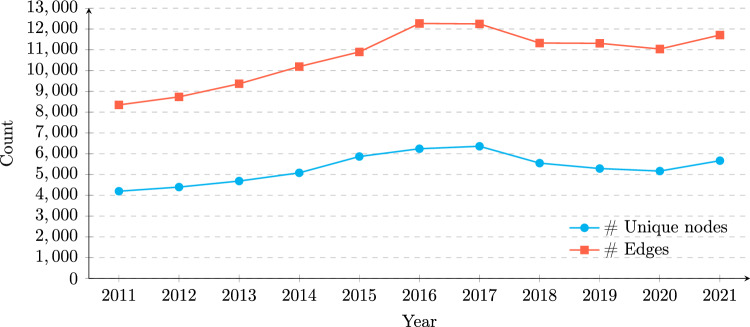
Change in the number of unique nodes and edges over time (2011–2021)

The numbers of nodes (GPs and LPs) and edges (the investment relationships between GPs and LPs) of each year are shown in Fig. 2. The annual node counts include LPs who made commitments during the respective year and GPs managing funds with commitments from LPs in the same year. On average, the number of LP nodes is twice the number of GP nodes.

Attributes of LPs include geographical preferences, strategy preferences, and industry preferences. Attributes of GPs include geographical focus, strategies, and industries. To be consistent, we treated both geographical preferences and geographical focus as region preferences. The regions we consider here are 23 sub-divisions within continents, such as “Central America”, “West Europe”, and “North Africa”. Industries include 13 industry groups, such as “Diversified”, “Clean Technology”, and “Information Technology”. For strategies, we follow the categorisation of Preqin (2025a). In principle, other relevant attributes, such as commitment size and assets under management (AUM), could provide further insights. However, their inclusion presents additional challenges, primarily due to the presence of missing data. Addressing this issue is beyond the scope of the current study and is left for future research.

Following Preqin’s categorisation of private capital in their database, we do not distinguish venture capital from private equity because “[t]echnically, venture capital is a lighter version of private equity” (Wang 2022). We distinguish between the networks of private equity including venture capital, private debt, real estate, natural resources, and infrastructure, and the hybrid network of all these asset classes together in one network.

To focus mainly on recent data, we model the networks of 2016–2021 while considering the impacts of the previous networks of 2011–2015 on tie formation in the following years. In modelling temporal networks, the specification of the node set eligible for tie formation matters for the baseline probability of establishing a tie. We define the networks in the respective asset classes to include all GPs and LPs who engaged in the network at any point between 2016 and 2021. This definition excludes GPs and LPs who were only active before 2016 or after 2021 but is otherwise inclusive and offers GPs and LPs active at one time in the network the opportunity to develop ties in other years as well. The numbers of GPs and LPs in the networks of each asset class and the hybrid network are shown in Table 1. It is worth noting that a GP may manage funds across various asset classes, and thus can appear in multiple networks. Similarly, an LP can also appear in multiple networks, as it may make commitments to funds in different asset classes. In some cases, GPs may also act as LPs in the same or different funds. This dual role can arise in situations where GPs choose to invest their own capital or contribute to other funds they manage, either as a strategic move or to demonstrate alignment with the interests of LPs. In our networks, we account for this by treating GPs as potentially having both roles, depending on the context. While this introduces some complexity in modelling, it reflects the reality of investment dynamics where GPs, as stakeholders in their own funds, may commit capital alongside external LPs.

**Table 1:** Number of GPs and LPs

Asset class	# GPs	# LPs
Hybrid (= all classes)	7153	7669
Private equity	5977	7236
Real estate	718	4192
Private debt	1014	3998
Natural resources	1126	4978
Infrastructure	559	4107

**Fig. 3:**
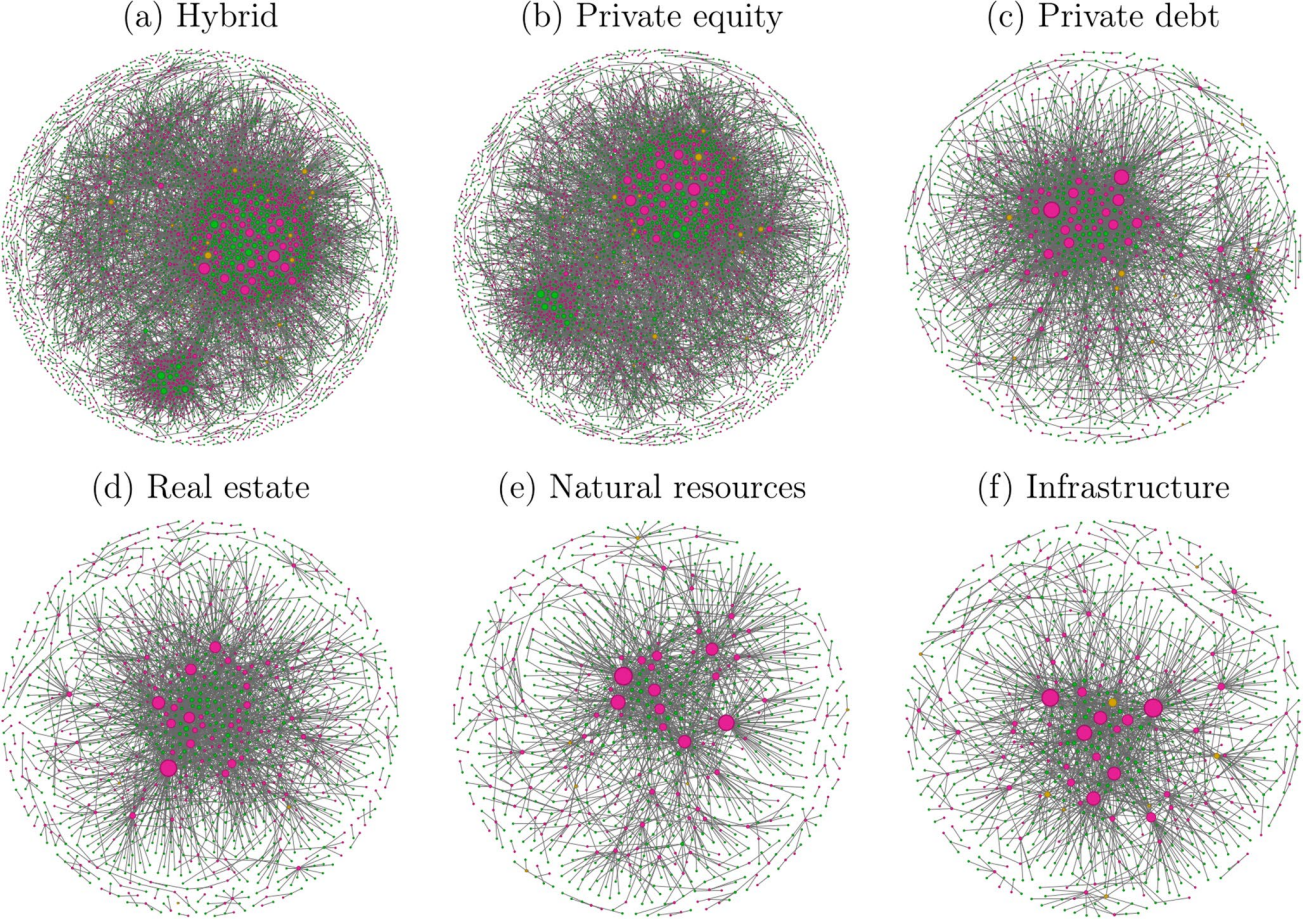
Graphs of the bipartite networks of each asset class in 2021 with a force-directed layout (Fruchterman and Reingold 1991). Node size is proportional to the degree (number of connections) within its respective network. Pink nodes represent GPs, green nodes represent LPs, and orange nodes represent GPs who are also LPs. Isolated nodes from the 2016–2020 dataset used for modelling are excluded from the visualisation.

To provide a first overview of network structures for the different asset classes, we visualise them using a force-directed layout (Fruchterman and Reingold 1991) in Fig. 3, taking the networks of 2021 as examples.

## The temporal exponential random graph model

A network model is required in order to drop the assumption that observations are independent and identically distributed (i.i.d.). Temporal exponential random graph models (TERGM) were estimated to model the network formation process (Hanneke and Xing 2010; Leifeld et al. 2018). The TERGM is a panel-data variant of the exponential random graph model (ERGM), which is often used to infer the network formation process in a single, cross-sectionally observed network (Hunter et al. 2008b; Krivitsky et al. 2023; Robins et al. 2007; Wasserman and Pattison 1996). The ERGM is a generalisation of logistic regression to multivariate observations with endogeneity among the observations of the dependent variable. The endogeneity in the network is modelled explicitly by including a vector of sufficient statistics $\varvec{h}$ to operationalise the dependence among dyads. The sufficient statistics in $\varvec{h}$ also contain effects of exogenous covariates $\varvec{X}$. In the TERGM, these sufficient statistics can be formed not only over the current network in a discrete sequence of networks but also over past networks in the sequence. Following the exposition in Leifeld et al. (2018), the joint likelihood of a series of observed networks $N^1, \ldots , N^T$ is defined as

1 $$\begin{aligned} & \mathcal {L} \left( N^{K+1}, \ldots , N^T \vert N^1, \ldots , N^K, \varvec{\theta }, \varvec{X} \right) \nonumber \\ & \quad = \prod _{t = K + 1}^T \frac{\exp \left( \varvec{\theta }^\top \varvec{h} \left( N^t, N^{t-1}, \ldots , N^{t-K}, \varvec{X} \right) \right) }{\sum _{N^{*} \in \mathcal {N}^t} \exp \left( \varvec{\theta }^\top \varvec{h} \left( N^{*}, N^{t-1}, \ldots , N^{t-K}, \varvec{X} \right) \right) }. \end{aligned}$$

The probability of a single network *N* at time *t* in the sequence, $N^{t}$, is the normalised, weighted sum of the vector of sufficient statistics *h* over the current and past networks, going back to $t-K$ from the perspective of the current time point *t*. The joint probability is the product of this probability over the different time points $K+1, \ldots , T$. Each *h* statistic can operationalise theory, for example by introducing covariates for the nodes and ties in the network at any point in time or by specifying counts of sub-graph products within or across time points, such as the number of ties, stars, or four-cycles. Here, we specify $K=5$ to let dyads in the current network $N^{t}$ depend on dyads in the previous five networks in the sequence, $N^{t-5}, \ldots , N^{t-1}$ and on dyads within $N^{t}$, as per Equations 9–11 below. This means that only data of the period 2016–2021 are included on the left-hand side of the model equation. We chose $K=5$ due to the sparsity of the networks and test if a tie in any of the past five years leads to an increased chance of a tie in the current year. This choice of *K* balances the trade-off between having fewer networks left to model (with greater *K*) and underestimating temporal dependence (with smaller *K*).

ERGMs and TERGMs can be estimated using Markov Chain Monte Carlo Maximum Likelihood Estimation (MCMC–MLE), Maximum Pseudolikelihood Estimation (MPLE), and Bayesian estimation (Leifeld et al. 2018). MCMC–MLE or Bayesian estimation is usually recommended for ERGM and TERGM estimation because they are unbiased. However, we chose MPLE for two reasons: Firstly, the size of the network at each time point is prohibitively large for MCMC–MLE or Bayesian estimation in terms of computation time. Secondly, $N^{K+1}, \ldots , N^T$ are conditionally independent observations of networks due to the temporal Markov dependence structure outlined in Equation 1. Thus, the pseudolikelihood estimator can be regarded as a composite likelihood estimator (Lindsay 1988), which is consistent with an increasing number of time points or network size (Desmarais and Cranmer 2012b). To mitigate the MPLE bias in the multiple hypothesis test, we employ Desmarais and Cranmer’s temporal bootstrap for TERGMs to generate confidence intervals around the estimates (Desmarais and Cranmer 2012b; Leifeld et al. 2018) using 100 bootstrap replications. This procedure draws temporal samples with replacement from the $T - K$ networks and repeats MPLE estimation to obtain a sampling distribution, leveraging the conditional independence of the networks implied by the temporal Markov structure. Confidence intervals are then generated from the 0.025 and 0.975 quantiles of this distribution. In some cases, this can lead to a point estimate falling outside the bootstrap confidence interval, since the point estimate is the observed pseudolikelihood coefficient without any bootstrap correction. More bootstrap samples would be desirable, but given the computational feasibility, only limited numbers of replications could be realised. We expect no significant differences with more replications but note that the bootstrap confidence intervals might become more accurate with a longer sequence of networks (Leifeld et al. 2018).

TERGMs require temporal homogeneity of coefficients because a single parameter is estimated per *h* statistic. Accommodating networks of vastly different sizes in the panel dataset may violate this assumption and lead to parameter heterogeneity. Schweinberger et al. (2020, 655) suggest possible remedies for ERGM estimation with multiple networks of different sizes. The TERGM presented here does not require any adjustments for network size because the networks have similar intrinsic sizes as shown in Fig. 2 and identical matrix dimensions as shown in Table 1. Shalizi and Rinaldo (2013) furthermore show that ERGM inference on subgraphs yields inconsistent estimates for the population network (“non-projectivity”). Schweinberger et al. (2020) show that consistent ERGM estimation for super-population inference does not require projectivity. Here, we estimate separate TERGMs for subgraphs induced by different asset classes. While non-projective in principle, we first explore whether these subgraphs display similar effects and then proceed by estimating the full (“hybrid”) TERGM over all asset classes. Non-projectivity poses no problem for our analysis because we do not infer the generative process of the hybrid network or LP–GP commitments in the super-population using estimates from the subgraphs.

**Fig. 4:**
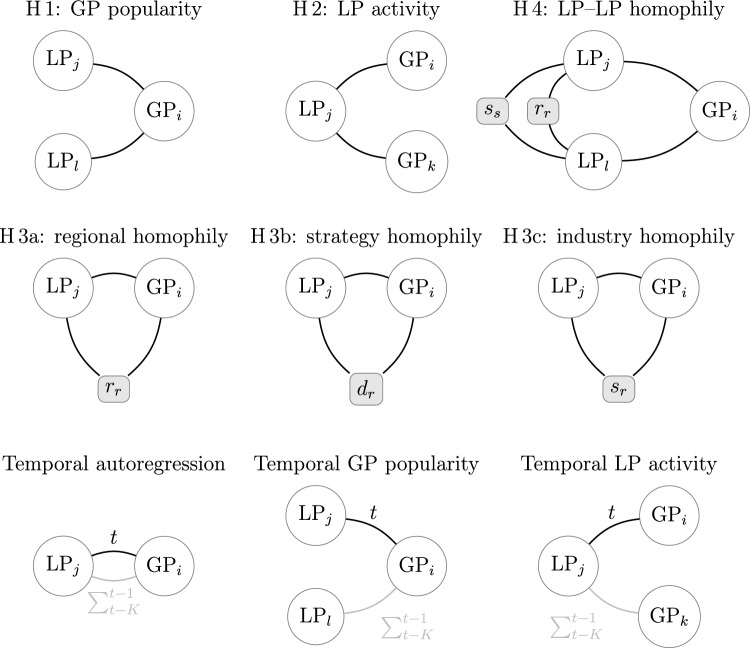
Illustration of the four hypotheses. First row: H 1, H 2, and H 4 represent structural effects implying dyadic dependence. Second row: H 3a, H 3b, and H 3c are dyadic effects based on exogenous node covariates, highlighted as shaded rectangles with rounded corners. Third row: Temporal effects, with ties in the previous five years coloured in grey. LP_j_ or LP_l_ is limited partner *j* or *l*, GP_i_ or GP_k_ is general partner *i* or *k*, $r_r$ is region *r*, $d_r$ is industry *r*, $s_r$ is strategy *r*, and $s_s$ is strategy *s.*

In the TERGM, we operationalise the hypotheses using the following *h* statistics. Indices *i*, *k* denote GPs; indices *j*, *l* denote LPs; and indices *r*, *s* denote regions, industries, or strategies. $N_{ij}$ refers to a specific GP–LP dyad as a cell in the bipartite network matrix at the current time *t*. *R*, *D*, *S* are binary matrices indicating the region, industry, and strategy preferences for GPs (matrix superscript GP) and LPs (superscript LP), with GP or LP names in the rows *i*, *j*, *k*, or *l* and different regions, industries, and strategies in the columns *r* or *s*. $[\ldots ]$ are Iversen brackets indicating 1 if the condition inside the square brackets is true and 0 otherwise. The network statistics corresponding to these hypotheses are also illustrated in Fig. 4.

H 1 counts the two-stars centred around GPs (“popularity”):

2 $$\begin{aligned} h_\text {popularity}(N^t) = \sum _i \sum _j \sum _l N_{ij}^t N_{il}^t [j \ne l] \end{aligned}$$

H 2 counts the two-stars centred around LPs (“activity”):

3 $$\begin{aligned} h_\text {activity}(N^t) = \sum _i \sum _j \sum _k N_{ij}^t N_{kj}^t [i \ne k] \end{aligned}$$

H 3a operationalises geographical/regional homophily between LPs and GPs:

4 $$\begin{aligned} h_\text {regional LP--GP homophily}(N^t, R) = \sum _i \sum _j \sum _r N_{ij}^t R_{ir}^\text {GP} R_{jr}^\text {LP} \end{aligned}$$

H 3b operationalises strategy homophily between LPs and GPs:

5 $$\begin{aligned} h_\text {strategy LP--GP homophily}(N^t, S) = \sum _i \sum _j \sum _r N_{ij}^t S_{ir}^\text {GP} S_{jr}^\text {LP} \end{aligned}$$

H 3c operationalises industry homophily between LPs and GPs:

6 $$\begin{aligned} h_\text {industry LP--GP homophily}(N^t, D) = \sum _i \sum _j \sum _r N_{ij}^t D_{ir}^\text {GP} D_{jr}^\text {LP} \end{aligned}$$

All three preference homophily sub-hypotheses are formulated as main and quadratic effects to capture diminishing marginal utility in the number of regions, industries, and/or strategies LPs and GPs share for forming deals. We expect positive main effects and negative quadratic coefficients in line with diminishing marginal utility. Including a squared effect in addition to the main effect is common practice in econometrics to model non-linear relationships, in this case because the effect of going from one to two shared industries or strategies is greater than, say, the effect of going from five to six shared industries or strategies.

H 4 operationalises regional and strategic homophily between LPs and other LPs around the same GPs:

7 $$\begin{aligned}&h_\text {LP--LP homophily}(N^t, R, S) \nonumber \\&\quad = \sum _i \sum _j \sum _l \sum _r \sum _s N_{ij}^t N_{il}^t [j \ne l] [R_{jr}^\text {LP} R_{lr}^\text {LP}> 0] [S_{js}^\text {LP} S_{ls}^\text {LP} > 0] \end{aligned}$$

Several control variables are included. The number of edges acts as an intercept:

8 $$\begin{aligned} h_{\text {edges}}(N^t) = \sum _{i} \sum _{j} N_{ij}^t \end{aligned}$$

We assessed the need for including a time trend but found that the intercept shows very little variation over time and opted for a single edges term.

Basic temporal dependence is captured using a lagged variable approach (“positive autoregression”, Leifeld et al. 2018), counting how many edges from the previous five years ($t - 5, \ldots , t - 1$) carry over to the next year (*t*):

9 $$\begin{aligned} h_{\text {autoregression}}(N^t, \dots , N^{t-K}) = \sum _{i} \sum _{j} N_{ij}^t \left( \sum _{\kappa = 1}^{K} N_{ij}^{t - \kappa } \right) \end{aligned}$$

For example, whether a tie occurs between an LP and a GP in 2018 is modelled as a function of the number of ties between the respective LP and GP from 2013 to 2017.

We also include temporal versions of GP popularity and LP activity in addition to the contemporaneous versions of the statistics in H 1 and H 2. Temporal popularity captures if LPs connect at higher rates to GPs who were popular in the previous five years,

10 $$\begin{aligned}&h_\text {temporal popularity}(N^t, \dots , N^{t-K}) \nonumber \\&\quad = \sum _i \sum _j N_{ij}^t \left( \sum _l \sum _{\kappa = 1}^{K} N_{il}^{t - \kappa } [j \ne l] \right) , \end{aligned}$$

and temporal activity tests if previously active LPs carry their tendency to form ties over to the present year,

11 $$\begin{aligned}&h_\text {temporal activity}(N^t, \dots , N^{t-K}) \nonumber \\&\quad = \sum _i \sum _j N_{ij}^t \left( \sum _k \sum _{\kappa = 1}^{K} N_{kj}^{t - \kappa } [i \ne k] \right) . \end{aligned}$$

Together, the TERGM treats these sub-graph counts as local processes that jointly produce the temporally evolving aggregate network structure. The estimated coefficients can be interpreted as increases in the log-odds ratios of establishing a tie when the respective covariate, or change statistic, is increased by one unit (e. g., when the number of shared industries between a GP and an LP in H 3b, $D_{ir}^\text {GP} D_{jr}^\text {LP}$, increases by a count of 1).

## Findings

The TERGM results are shown in Table 2. The positively significant coefficients of commitments from two LPs to one GP demonstrate the co-commitment dynamics within the LPs’ and GPs’ network. It is evident that networks tend to exhibit a higher prevalence of co-commitments than what would be expected by chance alone. The co-commitment behaviour could be attributed to shared interests, synergistic investment strategies, or mutual trust among LPs and GPs within the network. This finding is also compatible with the herd behaviour observed in the finance literature. Furthermore, the positive and statistically significant coefficients on LP activity confirm that LPs tend to diversify their commitments across different GPs, reflecting a strategy of portfolio diversification. Together, these results suggest that LPs and GPs do not make investment decisions in isolation, but are influenced by broader patterns of collaborative investment within the network.

**Table 2:** TERGM results for the hybrid network and for the networks in each asset class

		Hybrid			Private equity			Real estate		
*Structural effects*										
Edges		-11.1145^*^ (-11.2004 -11.0413)			-11.1353* (-11.2358 -11.0441)			-10.0325* (-10.1475 -9.9041)		
Popularity	H 1	0.0335* (0.0311; 0.0423)			0.0303* (0.0279; 0.0364)			0.0307* (0.0272; 0.0380)		
Activity	H 2	0.0771* (0.0737 ; 0.0812)			0.0858* (0.0801; 0.0923)			0.1835* (0.1707; 0.2018)		
LP–LP homophily	H 4	-0.0021* (-0.0030 -0.0011)			-0.0010 (-0.0025; 0.0008)			-0.0036* (-0.0047 -0.0021)		
*Dyadic effects*										
Common regions	H 3a	0.1489* (0.1326; 0.1690)			0.1511* (0.1317; 0.1741)			0.0742* (0.0609; 0.0867)		
Common regions (sqr)	H 3a	-0.0048* (-0.0063 -0.0039)			-0.0044* (-0.0060 -0.0035)			-0.0013* (-0.0024 -0.0007)		
Common strategies	H 3b	0.2005* (0.1744; 0.2234)			0.1600* (0.1292; 0.1876)			0.1717* (0.1576; 0.1880)		
Common strategies (sqr)	H 3b	-0.0090* (-0.0102 -0.0076)			-0.0072* (-0.0086 -0.0059)			-0.0062* (-0.0075 -0.0051)		
Common industries	H 3c	1.0688* (1.0131; 1.1224)			1.1156* (1.0381; 1.1743)			0.6180* (0.5464; 0.6993)		
Common industries (sqr)	H 3c	-0.1161* (-0.1226 -0.1097)			-0.1176* (-0.1255 -0.1100)			-0.0555* (-0.0663 -0.0457)		
*Temporal effects*										
Positive autoregression		2.3684* (2.0871; 2.6337)			2.2827* (2.0177; 2.5389)			1.9607* (1.6127; 2.2567)		
Temporal popularity		0.0013 (-0.0007; 0.0031)			0.0044* (0.0017; 0.0061)			0.0146* (0.0104; 0.0160)		
Temporal activity		-0.0004 (-0.0013; 0.0002)			0.0005 (-0.0000; 0.0008)			0.0013* (0.0011; 0.0016)		

		Private debt			Natural resources			Infrastructure		
*Structural effects*										
Edges		-9.8223* (-9.9603 -9.6214)			-10.2269* (-10.4899 -9.9931)			-10.4696* (-10.7486 -10.2328)		
Popularity	H 1	0.0306* (0.0274; 0.0339)			0.0346* (0.0311; 0.0398)			0.0341* (0.0316; 0.0418)		
Activity	H 2	0.1742* (0.1690; 0.1835)			0.2311* (0.2204; 0.2442)			0.2407* (0.2208; 0.2562)		
LP–LP homophily	H 4	-0.0014 (-0.0030; 0.0022)			-0.0053*- (0.0066 -0.0039)			-0.0057* (-0.0074 -0.0046)		
*Dyadic effects*										
Common regions	H 3a	0.0927* (0.0574; 0.1171)			0.0641* (0.0378; 0.0857)			0.0772* (0.0423; 0.1144)		
Common regions (sqr)	H 3a	-0.0022* (-0.0032 -0.0009)			0.0000 (-0.0012; 0.0014)			-0.0004 (-0.0021; 0.0012)		
Common strategies	H 3b	0.1575* (0.1274; 0.1852)			0.0590* (0.0346; 0.0825)			0.0547* (0.0409; 0.0724)		
Common strategies (sqr)	H 3b	-0.0061* (-0.0074 -0.0048)			-0.0017* (-0.0023 -0.0011)			-0.0017* (-0.0023 -0.0012)		
Common industries	H 3c	0.6141* (0.5277; 0.6802)			0.8489* (0.8048; 0.9109)			0.9499* (0.8508; 1.0206)		
Common industries (sqr)	H 3c	-0.0576* (-0.0657 -0.0481)			-0.0721* (-0.0772 -0.0686)			-0.0763* (-0.0869 -0.0632)		
*Temporal effects*										
Positive autoregression		1.9121* (1.6673; 2.1414)			1.6681* (1.4341; 1.8847)			1.6785* (1.4150; 1.9149)		
Temporal popularity		-0.0080* (-0.0121 -0.0022)			0.0089 (-0.0020; 0.0164)			-0.0011 (-0.0109; 0.0037)		
Temporal activity		-0.0005 (-0.0010; 0.0001)			0.0004* (0.0001; 0.0006)			0.0001 (-0.0000; 0.0004)		

*Zero outside the 95% bootstrapped confidence interval

We observe significant impacts of common preferences on the formation of the investment network between LPs and GPs. The effects relating to common preferences are consistent across the different asset classes. The model for the hybrid network shows the aggregate effects for all asset classes and is consistent with the separate effects in the other models. Positively significant coefficients for the number of common regional preferences, combined with negatively significant coefficients for the quadratic number of such preferences, indicate that as the number of shared regional preferences between LPs and GPs increases, the odds of investments between them also increase, albeit with a diminishing rate. However, this trend in the changing rate does not apply universally. In the case of infrastructure and natural resources, the coefficients for the squared term for common regions are not significant, implying a linear trend.

In addition to the positive result regarding regional preferences in the hybrid, private equity, and private debt networks, we find a similar pattern for the numbers of common strategies and the numbers of common industries. As the number of common strategies or common industries between LPs and GPs increases, the probability of investments between them also rises, albeit with a diminishing rate. Notably, unlike regional preference, there is no exception for common strategies and common industries. The same finding applies to the networks representing all asset classes. These consistent patterns highlight the reliability of these findings across various aspects of LP–GP network formation.

The results also reveal the effect of shared preferences among LPs concerning regions and strategies on their investment choices within the same GP (“LP–LP homophily”). However, this effect is not statistically significant within the private debt network. When LPs exhibit the same preferences as another LP who has already invested in a particular GP, they are less likely to commit capital to the funds managed by the same GP. This suggests a degree of competition or a diversification strategy among LPs with similar investment preferences, leading them to explore alternative GPs.

The significantly positive coefficients of edges in the previous five years (positive autoregression) reveal the effects of previous investment relationships in shaping future investment ties within the LP–GP network. Investments made in previous years between GPs and LPs prove to be an important factor that significantly increases the likelihood of establishing future investment connections between the same entities. This persistence in investment relationships highlights the enduring nature of collaboration within this network over time. We find that temporal popularity of GPs, whereby past commitment connections of GPs in the last five years translate into a higher rate of GP commitments in the current year, varies across in asset classes. Specifically, this effect is significantly positive in private equity (the largest class), real estate, but negative in private debt, and not significant in the aggregated (“hybrid”) network over all asset classes, natural resources, or infrastructure. Similarly, temporal activity of LPs is only significant in two asset classes—real estate and natural resources. Together, these temporal controls show that popularity and activity as per H 1 and H 2 are predominantly short-term structural effects, with an inconsistent long-term horizon.

Table 3 summarises the tested hypotheses and estimated parameters. Overall, the results reveal the existence of overarching principles and dynamics that govern LP–GP interactions, transcending the boundaries of specific asset classes. Even when considering various asset classes separately, the underlying short-term structural characteristics and attribute homophily exhibit similarities to each other and the unified, hybrid network. This suggests that there are common threads that connect LP–GP networks in diverse investment domains.

**Table 3:** Summary of hypotheses and model outcomes

No.	Hypothesis	Model outcome
H 1	Popularity: Multiple LPs tend to engage in investments with the same GPs.	Supported. Significant positive coefficient for co-commitments to the same GP.
H 2	Activity: Individual LPs tend to diversify their investments across different GPs.	Supported. Significant positive coefficient for commitments from one LP to multiple GPs.
H 3	Preference homophily: LPs are more inclined to invest in funds managed by GPs who share common preferences with them.	Supported overall. Shared preferences (regions, strategies, industries) increase likelihood of ties, with diminishing returns. The deminishing returns are not significant in common regions for natural resources and infrastructure.
H 4	LPs with shared regional and strategy preferences are less likely to invest in the same GPs.	Partially supported. Significant negative effects in most asset classes; not significant for private equity and private debt.

**Fig. 5:**
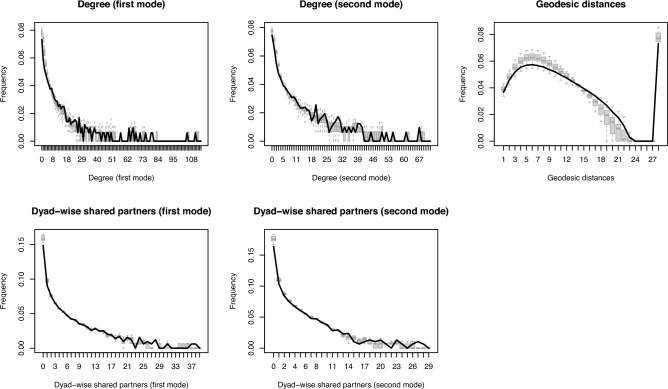
Goodness of fit assessment for the hybrid network model. Comparison of the observed network (black curves) to 100 simulated networks (grey boxplots) on several structural network indicators, showing a good alignment between model and simulations in terms of network structure.

Figure 5 shows how simulated networks based on the hybrid model in Table 2 compare to the observed network in 2021 on several key network metrics, following the goodness-of-fit (GOF) assessment procedure first proposed by Hunter et al. (2008a). We compare the degree distributions for GP and LP nodes, the distribution of geodesic distances (i. e., the shortest path distances between any two nodes), and the dyad-wise shared partner distributions for GP and LP nodes between the observed network (black lines) and 100 simulated networks (boxplots). We note that degrees of zero (“isolates”) are possible due to the construction of identical node sets for all time points as outlined in Sect. 3. As the observed network is close to the medians of the simulated distributions, the model captures the network properties well. This indicates that there is little bias in the coefficients and that the effects can be interpreted in meaningful ways. The overall small magnitude of deviations is in line with other published work using ERGMs and permits interpretation of the coefficients.

Appendix A presents similar GOF plots for networks corresponding to each asset class. The private debt network and the natural resources network fit particularly well, while the remaining networks are slightly misaligned on the geodesic distance distribution. All models slightly overestimate the number of GP–GP or LP–LP pairs that do not share an immediate partner but fit well overall.

**Fig. 6:**
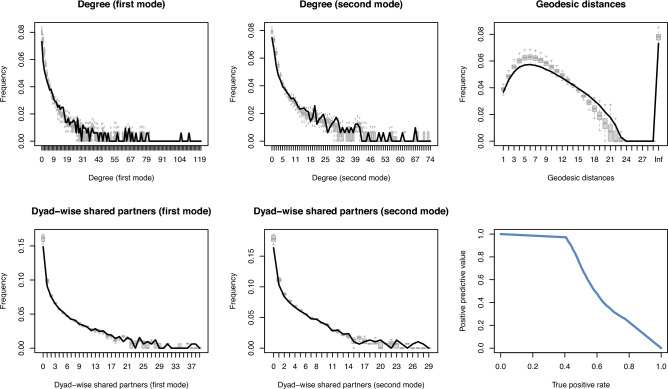
Goodness of fit assessment for the out-of-sample prediction of the hybrid network

A useful additional test of model performance is whether a model can predict well out of sample. In the context of TERGMs, it is common to simulate the last network in the temporal sequence (here: 2021) using a model estimated over all previous time points (here: 2016–2020) and compare the simulated networks to the held-out observed network (Leifeld and Cranmer 2019; Leifeld et al. 2018). We conduct such an out-of-sample performance check for the hybrid network. The estimated model overall displays similar coefficients as the model for the full sequence. As shown in Fig. 6, the model performs well overall out of sample, with the exception of a slight mismatch in the geodesic distance distribution. The endogenous out-of-sample model fit (Panels 1–5) is almost as good as the within-sample fit. The sixth panel of Fig. 6 shows the precision–recall curve, indicating useful precision (positive predictive value) and recall (true positive rate) in the prediction of future dyadic states.

**Fig. 7:**
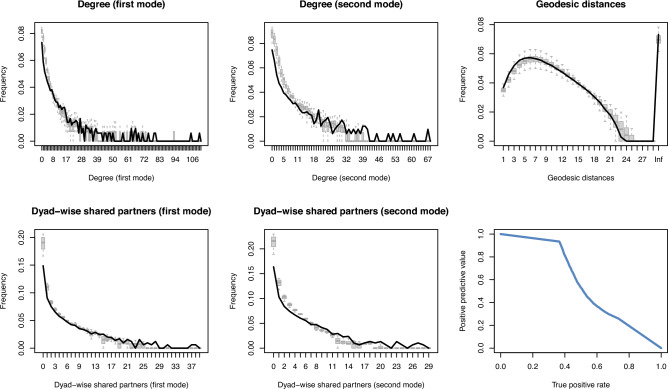
Goodness of fit assessment for the reduced model without structural terms in the hybrid network

We conduct several robustness checks for the structural and temporal specification. Firstly, we assess how much the structural effects contribute to the GOF by omitting the structural terms from the estimation in a separate specification. These omitted terms are “popularity”, “activity”, “LP–LP homophily”, “temporal popularity”, and “temporal activity”. These structural terms extend beyond one dyad (cf. Fig. 4). We then assess the resulting model fit, as shown in Fig. 7. The reduced model fits worse across most aspects, such as the degree distributions (for both modes) and dyad-wise shared partners (for both modes)—except for a marginal improvement in the geodesic distance distribution.

**Fig. 8:**
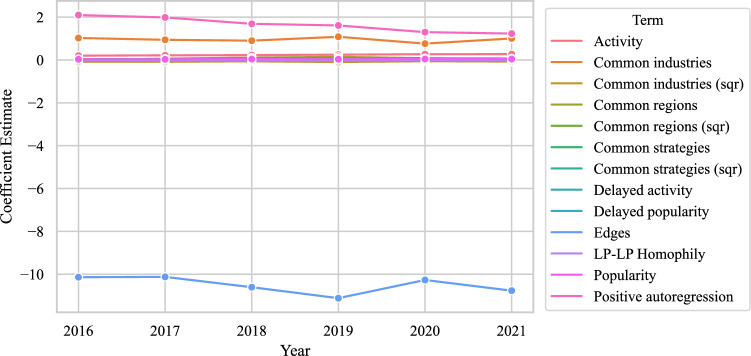
Temporal stability of coefficient estimates in separate cross-sectional ERGMs for the infrastructure network

Secondly, we assess the temporal specification using several robustness checks. We estimate six separate non-temporal ERGMs of the infrastructure network (for computational reasons) using MPLE to assess the temporal stability of the coefficients. As shown in Fig. 8, the results confirm that the coefficients remain stable over time. This backs our choice of a single intercept in the form of an edge count instead of time fixed effects or the inclusion of a time trend. In an additional robustness check (not documented here), we estimated a model with an edges term for the baseline log odds of establishing a tie in 2016 and fixed effects for years 2017–2021; none of these year effects showed significant deviations from the baseline edges term or changed the other coefficients significantly. We have thus captured the temporal specification adequately.

## Discussion

The findings of this study shed light on several significant aspects of the LP–GP investment network, unveiling the dynamics that govern this complex financial ecosystem. The limited availability of public data and the relatively smaller market size of certain private capital asset classes have historically constrained research in this field, which has predominantly focused on venture capital and private equity. Our results address this gap by encompassing a broader spectrum of asset classes, leveraging comprehensive industry-generated data.

A prominent insight from this study is the consistency of underlying investment dynamics across diverse private capital asset classes. Our findings reveal that LP–GP networks, whether in private debt, real estate, natural resources, or infrastructure, share common structural characteristics and behavioural drivers. Notably, the significant co-commitment tendencies and the strong homophily effects based on shared preferences—regions, strategies, and industries—emerge as near-universal patterns across all examined networks. This homogeneity suggests that despite differing market conditions and asset-specific nuances, fundamental principles, such as trust, alignment of investment objectives, and collaborative decision-making, dominate LP–GP interactions.

Interestingly, although minor deviations exist—such as the non-significance of the diminishing returns effect for common regional preferences in infrastructure and natural resources—the overall architecture of these networks remains fundamentally stable. This cross-asset consistency suggests that LPs and GPs navigate their investment decisions within a shared institutional framework, shaped by market conventions, regulatory environments, and informational asymmetries that transcend individual asset classes.

The trend of co-commitments can also be explained by the observation made by Hochberg et al. (2015), who suggested that venture capital firms form partnerships driven by the desire for resource accumulation. This notion of resource accumulation can be extended to other asset classes within the private capital domain.

The significantly positive effect of past investments should be related to collaboration and trust, which were suggested by Freiburg and Grichnik (2012). This effect also reflects the stickiness of the private capital market, which is driven by factors such as the illiquidity of investments and the unique, non-replicable skills of fund managers (Ljungqvist and Richardson 2003). Kaplan and Schoar (2005) argue that persistence in fund relationships stems from asymmetric information about GP skill, making repeated commitments a rational choice for LPs aiming to avoid adverse selection.

Overall, we observed that popularity and activity effects are short-term and play out mostly within a given year. The positive temporal popularity effects found in private equity and real estate supports the concept of cumulative advantage, whereby prior success enhances the likelihood of attracting future commitments. In this context, past popularity may serve as a proxy for perceived GP quality, reinforcing path-dependent dynamics in LP–GP relationships.

Importantly, these dynamics may be further amplified by institutional behaviours within the industry. For instance, consultants and other intermediaries often guide LPs towards established or previously successful funds, effectively channelling capital in line with prior popularity. Additionally, LPs may strategically follow commitments made by other institutions in order to reduce due diligence costs or to leverage the research efforts of their peers, thereby contributing to herding effects.

In private debt, however, the negative effect of temporal popularity suggests that LPs may intentionally avoid crowded funds, possibly as a means of preserving bargaining power or accessing less saturated opportunities. This behaviour aligns with concerns about capacity constraints and diminishing marginal returns in oversubscribed funds. It may also reflect market dynamics unique to private debt, where issuers that previously offered attractive terms (i. e., higher rewards for the same level of risk) can leverage increased demand to reduce those rewards in subsequent fundraising cycles. In such cases, LPs—facing limited bandwidth and resource constraints—may strategically shift away from previously popular GPs to seek more favourable risk–return profiles elsewhere. This divergence implies different levels of sensitivity to fund saturation and bargaining power across asset classes.

Moreover, temporal activity was positive in natural resources but negative in real estate, suggesting different investment dynamics. The consistency in natural resources may reflect a more stable, long-term investment approach tied to physical assets and extractive cycles, whereas the cyclicality of real estate markets may encourage more tactical, timing-driven allocations.

While we made efforts to ensure data accuracy, limitations in data quality should be acknowledged. Firstly, although it is common for funds to secure commitments from LPs in the year they are established, commitments often continue beyond that year. Our analysis captures only those commitments made in the fund’s vintage year and therefore omits subsequent commitments. This exclusion may introduce a systematic bias—particularly toward re-up capital—as first-year commitments are typically dominated by existing LPs familiar with the GP and more likely to anchor the first close. With fundraising cycles having lengthened in recent years, and a declining proportion of capital now secured in the first close (Preqin 2025b), our data may underrepresent new LP relationships formed later in the fundraising process. As a result, the observed strength of re-up patterns in our findings may be overstated, and the dynamics of broader LP–GP matching throughout the full fundraising period may not be fully captured. Secondly, funds lacking vintage year data were excluded from the dataset, along with the associated engaged LPs and GPs. However, this is not a significant concern as only 0.9% of funds are affected by missing year data.

One of the theoretical contributions of this study is a saturation mechanism in popularity effects, where the marginal benefit of herding may taper off once a GP attracts disproportionate attention. In our model, such non-linear dynamics are captured by the complex interplay of structural network effects, including two-star terms for popularity and activity. This choice reflects a parsimonious approach that captures the baseline tendency for LPs to cluster around popular GPs and aligns with standard practices in network modelling. However, we acknowledge that this non-linear specification may not explicitly capture the theorised diminishing marginal returns and rather models the marginal rate of additional popularity clustering (observed in Fig. 5) as a result of the interaction of popularity with activity and other network effects. Future research could explore advanced model specifications distinguishing between convex and concave popularity effects (Hunter 2007), micro-level interpretation of popularity (Desmarais and Cranmer 2012a), or advanced hypothesis testing frameworks such as Bayes factors (Mulder et al. 2024) to test for inflection points in popularity effects more explicitly. We also note that the saturation mechanism serves a dual purpose in our framework: It not only enriches the theoretical understanding of popularity (H 1) but also motivates the diversification behaviour captured in the activity hypothesis (H 2).

Future research should also address computational limitations in (temporal) exponential random graph models and their implementation. We estimated a TERGM with MPLE and bootstrapped confidence intervals because MCMC sampling of large networks such as the ones considered here is prohibitively slow. Available TERGM implementations also require storage of the network data in matrix form, which has high memory requirements during the estimation and may not be feasible in future research when new years are added to the model. Research in computational statistics should consider implementing these models using edge lists and possibly in conjunction with memory-based graph databases to address the size and memory issue. It should also consider computationally more efficient ways to estimate models when the network is large, for example using appropriate sampling strategies within the network (Stivala et al. 2020), graph limits (He and Zheng 2013), MPLE with approximations of the inverse Hessian matrix (Schmid and Hunter 2023), bootstrapping (Schmid and Desmarais 2017), efficient pooling over multiple networks (Yin and Butts 2022), and numerical gains through local constraints (Vallarano et al. 2021). Here, we used one such technique (Desmarais and Cranmer 2012b), but a comparison between methods is needed to understand the implications of different choices for computation time, memory footprint, unbiasedness, efficiency, and applicability to temporal networks better.

Applied research should attempt to leverage the insights generated by our analysis to predict future investment. The TERGM is a likelihood-based model and estimates coefficients. This property can be exploited to make model-based predictions out of sample for the benefit of market forecasts. Alternatively, recent advances in graph neural networks could be leveraged for tie prediction in future years (Zhang and Chen 2018). For increased precision and, ultimately, market adoption, however, the data need to be collected in real time and with more accurate time stamps to learn more accurately from past network dynamics for timely forecasting.

In conclusion, this study provides a comprehensive understanding of the LP–GP investment network, transcending the boundaries of specific asset classes. The common trends and dynamics identified in this research offer valuable insights for future studies seeking to extend findings from one asset class to another. The overarching principles governing LP–GP interactions provide a foundation for a more holistic understanding of the private capital landscape, offering practical insights to address the matching problem between GPs and LPs. This study opens new avenues for research in the realm of private capital, encouraging scholars to explore the dynamics of LP–GP networks across diverse investment domains.

## Figures and Tables

**Fig. 9:**
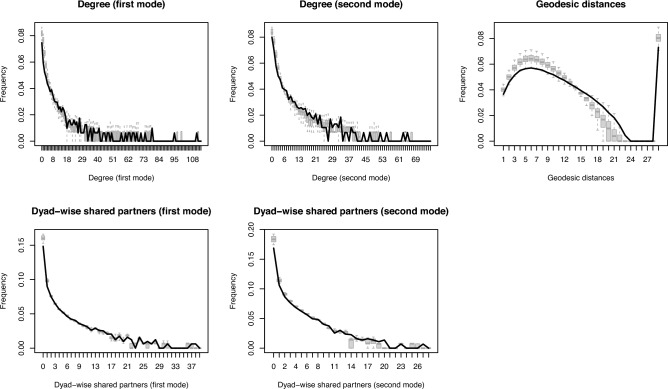
Goodness of fit assessment for the private equity network

**Fig. 10:**
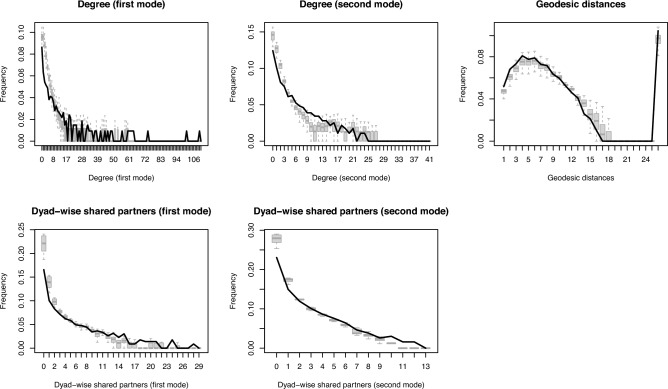
Goodness of fit assessment for the private debt network

**Fig. 11:**
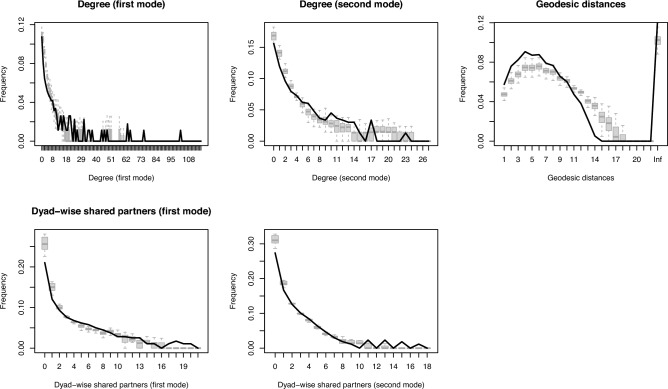
Goodness of fit assessment for the real estate network

**Fig. 12:**
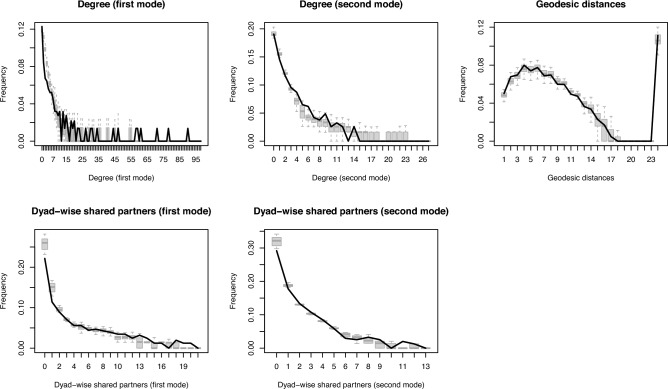
Goodness of fit assessment for the natural resources network

**Fig. 13:**
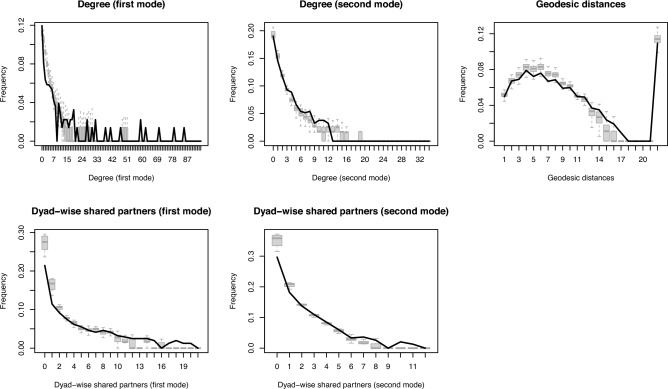
Goodness of fit assessment for the infrastructure network

## Data Availability

The manuscript analyses data collected by and supplied to the authors by Preqin Ltd. as part of a Knowledge Transfer Partnership. Preqin, which is a part of BlackRock Inc., granted consent for publication in 2024. Neither Preqin nor BlackRock have influenced the research results. Data enquiries can be addressed to Dr Yuanyuan Shang at Preqin.

## A Appendix: Goodness-of-fit assessment

See appendix Figs. 9, 10, 11, 12 and 13.

## Abbreviations

CEO: Chief executive officer
GOF: Goodness-of-fit
GP: General partner
IRR: Internal rate of return
LP: Limited partner
MCMC-MLE: Markov Chain Monte Carlo maximum likelihood estimation
MPLE: Maximum pseudo-likelihood estimation
NAV: Net asset values
TERGM: Temporal exponential random graph model
VC: Venture capital
